# A network approach to relationships between cannabis use characteristics and psychopathology in the general population

**DOI:** 10.1038/s41598-022-11092-0

**Published:** 2022-05-03

**Authors:** Linda T. Betz, Nora Penzel, Joseph Kambeitz

**Affiliations:** 1 Department of Psychiatry and Psychotherapy, Faculty of Medicine and University Hospital of Cologne, University of Cologne, Cologne, Germany; 2 Department of Psychiatry and Psychotherapy, Ludwig-Maximilian-University, Munich, Germany; 3 Group of Psychiatric Neuroscience, Department of Basic Medical Sciences, Neuroscience and Sense Organs, University of Bari ‘Aldo Moro’, Bari, Italy

**Keywords:** Risk factors, Epidemiology, Lifestyle modification, Preventive medicine, Drug regulation, Psychosis, Depression

## Abstract

Cannabis use characteristics, such as earlier initiation and frequent use, have been associated with an increased risk for developing psychotic experiences and psychotic disorders. However, little is known how these characteristics relate to specific aspects of sub-clinical psychopathology in the general population. Here, we explore the relationships between cannabis use characteristics and psychopathology in a large general population sample (*N* = 2,544, mean age 29.2 years, 47% women) by employing a network approach. This allows for the identification of unique associations between two cannabis use characteristics (lifetime cumulative frequency of cannabis use, age of cannabis use initiation), and specific psychotic experiences and affective symptoms, while controlling for early risk factors (childhood trauma, urban upbringing). We found particularly pronounced unique positive associations between frequency of cannabis use and specific delusional experiences (persecutory delusions and thought broadcasting). Age of cannabis use initiation was negatively related to visual hallucinatory experiences and irritability, implying that these experiences become more likely the earlier use is initiated. Earlier initiation, but not lifetime frequency of cannabis use, was related to early risk factors. These findings suggest that cannabis use characteristics may contribute differentially to risk for specific psychotic experiences and affective symptoms in the general population.

## Introduction

Prospective epidemiological studies have consistently reported an association between cannabis use and an increased risk for subsequent psychotic experiences and psychotic disorders^1–3^. However, only a minority of individuals who use cannabis will eventually develop a psychotic disorder. Thus, recent research efforts aim to identify aspects of exposure to cannabis that are particularly potent in increasing the risk for psychosis and psychotic experiences, including higher frequency and duration of use^4–7^ and initiation at a younger age^4,8–12^. Initiation of cannabis use at a young age may be particularly harmful as adolescence is a critical period of increased vulnerability to the effects of cannabis due to developmental and maturational processes in key areas of the brain^11,13–18^.

Prior investigations on the psychopathological effects of cannabis use characteristics have focused on broad mental health outcomes, such as diagnosis with a psychotic disorder or compound measures of psychopathology^1,8,9,19–22^. A first study found associations between earlier initiation of cannabis use and both positive and negative symptom dimensions of psychosis (i.e., distorted or excessive normal functions such as delusions, hallucinations, disorganized behavior vs. diminished or absent normal functions related to motivation and interest such as avolition, flattening of affect, and poverty of speech^23^), but not depressive symptoms in a large young-adult general population sample^9^. Conversely, in a nationally representative study of 19-year-olds in Greece, both lifetime frequency and earlier age of cannabis use initiation were associated with increases in psychotic clusters of hallucinations, paranoia, grandiosity, and first-rank symptoms, but not in dimensions of negative symptoms and depression^20^. In a third study, daily, compared to non-daily non-psychotic cannabis users, showed greater prevalence of symptom clusters of first-rank symptoms, hallucinations, and grandiosity, even after controlling for age of cannabis use initiation^22^. In a fourth study conducted in a large community sample of adolescents in Australia, higher frequency of cannabis use in the last year was associated with higher scores on subscales of perceptual abnormalities and magical thinking, but not with bizarre thinking and persecutory ideation^21^. Finally, in a population sample drawn from the UK Biobank, there was a dose-dependent relationship between frequency of cannabis use and psychotic experiences, particularly persecutory delusions^24^. Even though somewhat mixed regarding findings on negative symptoms, these studies overall suggest a certain specificity in the association between cannabis use characteristics and psychopathology in the psychosis continuum: earlier initiation and frequency of cannabis use do not appear to affect all symptom domains equally.

In line with this observation, there is increasing awareness that interactions between individual risk factors and symptoms may offer a nuanced insight into the etiology of psychosis^25–30^. More specifically, adopting a network perspective represents one promising approach to disentangle the multifaceted ways by which cannabis use characteristics relate to the occurrence of attenuated expressions of positive psychotic symptoms below the diagnostic threshold (also called *psychotic experiences*^31^). Network theory conceptualizes psychological behavior as a complex interplay between symptoms, biological, sociological, and environmental components^25,32,33^. Following this approach, the focus shifts from investigating broad outcomes, such as diagnosis with a psychotic disorder or sum scores of psychotic dimensions, to interactions between individual symptoms and other clinically relevant components, such as environmental risk factors^25,26,28,34^.

Typically, statistical network models based on cross-sectional data depict unique relationships between variables, representing the share of the association between two variables that remains after controlling for all other variables in the network^35^. This allows for a simultaneous analysis of all relationships that may be important in a network of connected phenomena. Hence, network models are a suitable choice to uncover the specific relationships in the context of distinct cannabis use characteristics, such as frequency and age of cannabis use initiation, that are typically not independent: earlier initiation of use is more likely to become longstanding^8^. Assessing either aspect in isolation, without controlling for the respective other, may likely overestimate its effect on individual aspects of psychopathology. Similarly, there is evidence that cannabis use and other early environmental risk factors for psychosis, such as childhood trauma and urbanicity, are not independent^7,25,36,37^. When examining the associations between cannabis use characteristics and individual symptoms, it is therefore important to take further available cannabis use characteristics and environmental risk factors into account to derive unique associations, i.e., associations that remain even after controlling for the other factors. Such joint modeling acknowledges the complex dependencies in environmental risk^29,37,38^. Likewise, expanding focus to domains of psychopathology beyond positive psychotic experiences has proven informative for a comprehensive account of the complex etiology of psychotic experiences and full-blown psychotic disorders^25,28,29,34,39–41^. For example, the mediating role of affective psychopathology in pathways from environmental risk factors to psychotic psychopathology is increasingly recognized^28,29,40,41^.

In the present work, we take a network approach to explore the unique relations between specific cannabis use characteristics, i.e., age of cannabis use initiation and lifetime cumulative frequency, a broad spectrum of psychotic experiences and affective psychopathology, as well as early environmental risk factors such as childhood trauma and urbanicity, in cannabis users of a large general population sample (i.e., those who reported having used cannabis at least once in their lifetime). With these analyses, we extend the existing literature on cannabis use characteristics and psychopathology in three ways. First, we investigate the associations between distinct cannabis use characteristics and individual aspects of psychopathology, avoiding binarized measures of cannabis use characteristics that may obscure important associations. Second, we take both the cumulative frequency and the age of cannabis use initiation into account. Third, we simultaneously model childhood trauma and urban upbringing as early environmental risk factors in the network. Using this approach, we can identify unique associations, i.e., which specific symptoms are related to cannabis use characteristics, after controlling for all other modeled symptoms, cannabis use characteristics, and environmental risk factors^42^.

## Method

### Sample

The data used in this study come from the National Comorbidity Survey (NCS)^43^, a collaborative epidemiological investigation based on a nationally representative, stratified, multistage, area probability sample of persons in the age range 15–54 in the non-institutionalized population of the 48 coterminous states of America designed to study the prevalence and correlates of psychiatric disorders between 1990 and 1992. Overall response rate was 82.4%, with a total of 8,098 participants. Informed consent was obtained from all participants. The NCS interview was administered in two parts. Part I was administered to all respondents and contained the core diagnostic interview, as well as a brief risk factor battery. A subsample of the original respondents (*N* = 5877) completed the additional NCS Part II survey that contained a more detailed risk factor battery and additional diagnostic assessments. The current study is based on respondents in the Part II subsample. We limited the Part II subsample to participants who reported any lifetime cannabis use and were aged 40 and younger at the time of assessment (*N* = 2624) to reduce the possibility to capture secondary psychosis related to (beginning) neurodegenerative disorders, and due to concerns about recall and reporting artifacts^45,46^. A full description of the NCS is available elsewhere^43^.

The original NCS data collection protocol was approved by the University of Michigan’s Internal Review Board (IRB). All procedures contributing to this work comply with the ethical standards of the relevant national and institutional committees on human experimentation and with the Helsinki Declaration of 1975, as revised in 2008.

## Measurements

### Psychopathology

A modified version of the Composite International Diagnostic Interview (CIDI)^43,47,48^ was used in the NCS. The CIDI is a non-clinician administered diagnostic interview developed jointly by the Alcohol, Drug Abuse, and Mental Health Administration (ADAMHA) and the World Health Organization (WHO) to facilitate psychiatric epidemiologic research^47^. Modifications of the CIDI for the NCS are described in detail in^48,49^ and all study materials can be retrieved from https://www.hcp.med.harvard.edu/ncs/Baseline_NCS.php. The psychosis screening section of the CIDI (Section K) contained 13 items related to psychotic experiences and beliefs, all of which were included in our analyses. To represent more general dimensions of psychopathology, we included 6 items from the lifetime mood and health behaviors screening section of the CIDI (Section B), including lifetime experiences of panic, anxiety, sadness, loss of interest, mania, and irritability. All items were responded to by all participants used for the present analyses (i.e., there was no skip-structure), using a simple “yes” or “no” response format. For details on these assessments, see Supplementary Table 1.

### Cannabis use characteristics

We included two available cannabis use characteristics derived from the Medication and Drugs module in the NCS: the age of cannabis use initiation (age at which cannabis was first used) and cumulative lifetime frequency of cannabis use, expressed as the total number of cannabis use occasions, which were coded in a binned format (1 or 2 times, 3 to 5 times, 6 to 10 times, 11 to 49 times, 50 to 99 times, 100 to 199 times, 200 or more times). For details on these assessments, see Supplementary Table 1.

### Early risk factors

To control for exposure to early environmental risk, we included childhood trauma and urban upbringing. Information on childhood trauma was derived from the Posttraumatic Stress Disorder (PTSD) module from the modified version of the CIDI^48^. In accordance with prior analyses in the NCS^50–52^, we selected five questions that represented (1) childhood neglect, (2) childhood physical abuse, (3) rape (before age 18), and (4) sexual molestation (before age 18). Items were scored with a “yes” or “no” response format. No explicit age limit was stated for “childhood” events (1, 2). Question 1 was used to represent childhood neglect. Again, following prior work in the NCS^51,52^, questions 2–4 were collapsed into a binary variable representing childhood abuse, which indicated if a participant had given a “yes” response to any of these questions. The variable urban upbringing reflected whether participants had been raised in a suburb or city during most of their childhood. For details on these assessments, see Supplementary Table 1.

### Covariate

To control for age-related links between cannabis use characteristics (e.g., older individuals having often used more) and psychopathology (e.g., some symptoms manifesting later than others), we additionally included age at assessment as a covariate in the network model.

## Data analytic strategy

We conducted all analyses using *R*, version 4.1.0^53^. Throughout, we considered a significance level of α < 0.05. Reporting complied with recently proposed standards for network analyses in cross-sectional data^54^. Data used in this study are available for public use via the Inter-university Consortium for Political and Social Research^44^. Code to reproduce the analyses is available at https://github.com/kambeitzlab/Network_Cannabis.

### Network estimation

Because the data contained continuous, ordinal, and binary variables, we chose an undirected mixed graphical model for network estimation^55,56^. Items assessed on ordinal scales without equal spacing have typically been treated as continuous in this particular modeling context in prior work (e.g., ^56,57^). Thus, lifetime cumulative frequency of cannabis use, age of cannabis use initiation, and age at assessment were treated as continuous variables, while the remaining items relating to early risk factors and psychopathology, coding the presence or absence of the respective factor, were treated as categorical variables in the estimation of the mixed graphical network model. In such a network, variables are represented by nodes, and edges between two nodes reflect the association between the corresponding variables that remains after controlling for all other variables under consideration. Edges can be interpreted as predictive effects, i.e., the share of the pairwise association between two variables that cannot be explained by any other variable in the network, also known as conditional dependence relation^35^. If two variables are independent conditioned on all other variables, no edge is drawn between them in the network. Estimation of mixed graphical models is based on a so-called pseudo-likelihood, node-wise regression approach^58^, where each variable is predicted by all other variables in a L_1_-regularized Generalized Linear Model (GLM) framework. The link function used in the GLM depends on the type of exponential family distribution of a given variable^59^ (in the present case, Gaussian distribution for the two continuous cannabis use-related variables and Bernoulli distribution for all remaining binary variables). This node-wise regression approach leads to two estimates for each edge weight that we combined using the “OR” rule, meaning that at least one edge weight estimate had to be non-zero in order to set the edge to be present in the network^56^.

L_1_-regularization ensures a high specificity of the edges in the network^60^. The optimal penalty parameter used in regularization was determined by minimizing the Extended Bayesian Information Criterion (EBIC^61^). The EBIC itself has a hyperparameter, γ, that governs the amount of regularization in the network; the higher γ, the more regularization is imposed, and the higher the possibility of false negatives edges in the network^61^. The findings reported in the main paper are based on γ = 0, ensuring maximal sensitivity^57,62^. Additionally, we systematically varied γ from 0 to 0.25 in steps of 0.05 to test the impact of the amount of regularization on our findings. We constructed the networks using the *R* package ‘mgm’, version 1.2–12^56^, and visualized them using the *R* package ‘qgraph’, version 1.6.9^63^. Of note, ‘mgm’ does currently not allow missing values. We therefore tested whether data were missing completely at random (MCAR) using the nonparametric test of homoscedasticity described by Jamshidian and Jalal^64^, as implemented in the *R* package ‘MissMech’, version 1.0.2^65^. If the MCAR assumption is met, removal of observations with missing data is expected to produce unbiased estimates of the parameters in the network model^66,67^.

For visualization, we manually placed the two nodes representing characteristics of cannabis use (age of initiation, lifetime cumulative frequency) in the center of the network, as these variables, and their association with symptoms, were the focus of the analysis. The positioning of the remaining nodes was determined using the Fruchterman-Reingold algorithm, placing more strongly connected nodes to the center and less connected nodes to the periphery of the network^68^. Additionally, we manually un-faded edges connected to the two nodes representing cannabis use characteristics. i.e., these edges were deliberately set opaque, while the other edges retained transparency depending on their respective edge weight^26^. The cut-value was set to 0, meaning that for plotting the network, no cut-off was used to curtail the scaling of edges in width and color saturation; rather, all edges were allowed to vary in width and color depending on their strength and sign (for details, see^63^).

Following recommended guidelines^42^, we employed the routine implemented in the *R* package ‘bootnet’, version 1.4.3^69^ to assess the stability and robustness of the estimated network structures with respect to proneness to sampling variation and dropping of cases with 1,000 bootstrap samples.

To assess the effect of sex on our results^29^, we estimated a moderated mixed graphical model^70^ via the *R* package ‘mgm’^56^. Here, we focused on moderation-effects of sex on network connections related to variables of cannabis-use characteristics, i.e., age of cannabis use initiation and cumulative lifetime frequency of cannabis use. We tested the stability of the results using 1,000 bootstrap samples.

## Results

### Sample characteristics

Of 2,624 participants, 80 (3.0%) had to be excluded due to missing values in the network variables of interest. Missing data met the MCAR assumption (*p* = 0.624), supporting a complete case analysis. The final sample thus comprised *N* = 2,544 participants, 47.0% percent of whom were women, with an average age of 29.2 years (*SD* = 6.5) at assessment. Mean age of cannabis use initiation was 16.7 years (*SD* = 3.2). On average, participants had consumed cannabis 11 to 49 times in their lives. Table 1 provides details on demographic characteristics of the sample. Lifetime prevalences of the modeled affective symptoms were as follows: *panic*: 35.4%, *anxious*: 52.6%; *sad*: 54.3%; *loss interest*: 50.2%; *irritable*: 36.0%; *manic:* 11.7%. Lifetime prevalences of the modeled psychotic experiences were as follows: *spying/following you:* 14.3%; *poison/hurt you:* 3.9%; *reading your mind*: 7.8%; *hear your thoughts*: 4.5%; *hear others thought*: 7.5%; *controlled by force*: 3.8%; *others stole thoughts*: 2.7%; *special messages/tv:* 2.7%; *hypnotized/magic/force:* 1.3%*; saw visions:* 9.0%; *heard noise/voice:* 8.6%; *smells/body odors*: 5.0%; *feelings in/on body*: 8.5%.

**Table 1:** Demographics of the study sample (*N* = 2,544).

Variable	
Sex, n (%)	Women: 1196 (47.0); Men: 1348 (53.0)
Age in years, mean (SD)	29.2 (6.5)
Education, n (%)	Less than high school: 385 (15.1); high school or equivalent: 857 (33.7); some college: 760 (29.9); college degree and beyond: 197 (7.7); no information: 345 (13.6)
Ethnicity, n (%)	White: 2081 (81.8); Black: 245 (9.6); Hispanic: 150 (5.9); Other: 66 (2.6); no information: 2 (0.1)
Immigration status, n (%)	U.S.-born: 2455 (96.5); foreign-born: 89 (3.5)
Age of cannabis use initiation, mean (SD)	16.7 (3.2)
Lifetime cumulative frequency of cannabis use, n (%)	1 or 2 times: 462 (18.2); 3 to 5 times: 329 (12.9); 6 to 10 times: 277 (10.9); 11 to 49 times: 438 (17.2); 50 to 99 times: 226 (8.9); 100 to 199 times: 182 (7.2); 200 or more times: 630 (24.8)
Time last used cannabis, n (%)	Past month: 348 (13.7); past six months: 234 (9.2); past year: 113 (4.4); more than a year ago: 1844 (72.5); no information: 5 (0.2)
Childhood abuse, n (% yes)	422 (16.6)
Childhood neglect, n (% yes)	116 (4.6)
Urban upbringing, n (% yes)	1181 (46.4)

### Network

**Figure 1:**
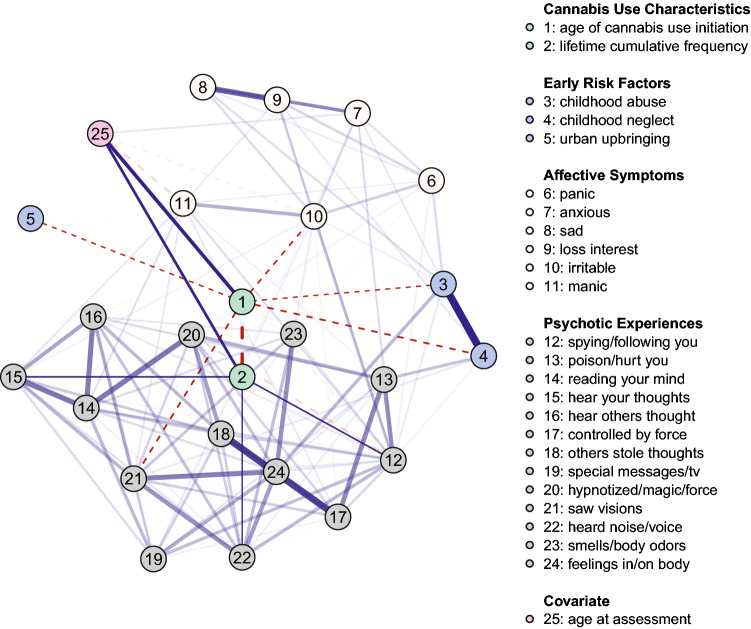
Network of cannabis use characteristics (age of cannabis use initiation, lifetime cumulative frequency of cannabis use), early risk factors, psychotic experiences, and affective symptoms (*N* = 2,544). Solid blue (dashed red) lines represent positive (negative) associations between variables and wider, more saturated edges indicate stronger associations. Given that the focus of the paper was to investigate the relations between the cannabis use characteristics and aspects of psychopathology, the edges connecting to the two relevant variables (age of cannabis use initiation, lifetime cumulative frequency of cannabis use) have been manually un-faded, i.e., these edges were deliberately set opaque, while the edges between the other nodes in the network retain transparency. Variable groups are differentiated by color.

Figure 1 depicts the network illustrating unique relationships between cannabis use characteristics, early environmental risk factors, as well as psychotic experiences and affective psychopathology (for the individual edge weights, see Table 2). Of 300 possible edges, 121 (40.3%) were retained in the regularized mixed-graphical model estimation, with a mean edge weight of 0.08. Results show that *age of cannabis use initiation* is negatively related to *saw visions* (*edge weight* (*w*) = *− *0.08), *irritable* (*w* = − 0.06), and early risk factors, including *childhood neglect* (*w* = − 0.08) and *abuse* (*w* = − 0.02) as well as *urban upbringing* (*w* = − 0.02). These results suggest that *earlier* initiation of cannabis use makes the positive endorsement of these variables more likely (e.g., younger age at first use of cannabis makes lifetime experiences of visual hallucinations more likely). *Lifetime cumulative frequency* showed positive links to *hear your thoughts* (*w* = 0.05), *spying/following you* (*w* = 0.04), and *heard noise/voice* (*w* = 0.02), indicating that the higher lifetime cumulative use, the more likely these experiences become. The covariate *age at assessment* shows positive links to both *age of cannabis use initiation* (*w* = 0.39) and *lifetime cumulative frequency of cannabis use* (*w* = 0.24), suggesting that the older the person was at assessment, the later they started consuming cannabis on average, and the more often they had cannabis consumed in their lifetime.

**Table 2:** Edge weights for the network shown in Fig. 1 (obtained via mixed graphical-model estimation).

	1	2	3	4	5	6	7	8	9	10	11	12	13	14	15	16	17	18	19	20	21	22	23	24	25
1	0	-0.35	-0.02	-0.08	-0.02	0	0	0	0	-0.06	0	0	0	0	0	0	0	0	0	0	-0.08	0	0	0	0.39
2	-0.35	0	0	0	0	0	0	0	0	0	0	0.04	0	0	0.05	0	0	0	0	0	0	0.02	0	0	0.24
3	-0.02	0	0	1.06	0	0.18	0.11	0.16	0	0.07	0	0.09	0	0	0	0	0	0	0	0	0	0	0	0.34	0
4	-0.08	0	1.06	0	0	0	0	0	0.10	0	0	0.06	0.30	0	0	0	0	0	0	0	0.11	0	0	0	0
5	-0.02	0	0	0	0	0	0	0	0	-0.03	0	0	0	0	0	0	0	0	0	0	0	0	0	0	0
6	0	0	0.18	0	0	0	0.15	0.09	0.20	0.24	0.07	0.06	0	0.03	0	0	0	0	0	0.04	0	0	0.03	0	0
7	0	0	0.11	0	0	0.15	0	0.38	0.31	0.18	0	0.13	0.07	0	0	0	0	0	0.04	0	0	0	0	0	0.12
8	0	0	0.16	0	0	0.09	0.38	0	0.74	0.17	0	0	0	0	0	0	0	0	0	0	0	0	0	0	0
9	0	0	0	0.10	0	0.20	0.31	0.74	0	0.19	0.09	0.13	0	0	0	0	0	0	0	0	0	0	0	0	0
10	-0.06	0	0.07	0	-0.03	0.24	0.18	0.17	0.19	0	0.39	0.28	0	0.02	0.07	0.03	0.04	0	0.08	0	0	0	0.09	0.03	-0.07
11	0	0	0	0	0	0.07	0	0	0.09	0.39	0	0.16	0.06	0.15	0.11	0	0.11	0	0	0	0.10	0	0	0	-0.17
12	0	0.04	0.09	0.06	0	0.06	0.13	0	0.13	0.28	0.16	0	0.53	0.21	0.04	0.11	0.20	0.2	0.32	-0.18	0.10	0.34	0.11	0.13	-0.07
13	0	0	0	0.30	0	0	0.07	0	0	0	0.06	0.53	0	0	0	0.18	0.59	0	0.13	0.38	0	0.08	0	0.10	0
14	0	0	0	0	0	0.03	0	0	0	0.02	0.15	0.21	0	0	0.67	0.64	0	0.28	0.34	0.66	0	0.07	0.17	0.17	0
15	0	0.05	0	0	0	0	0	0	0	0.07	0.11	0.04	0	0.67	0	0.44	0	0.26	0.18	0	0.36	0.14	0	0	0
16	0	0	0	0	0	0	0	0	0	0.03	0	0.11	0.18	0.64	0.44	0	0.03	0.34	0.11	0	0.38	0.29	0.11	0	0
17	0	0	0	0	0	0	0	0	0	0.04	0.11	0.20	0.59	0	0	0.03	0	0.94	0.07	0.20	0.24	0.02	0	0.26	0
18	0	0	0	0	0	0	0	0	0	0	0	0.20	0	0.28	0.26	0.34	0.94	0	0.24	0.47	0	0.24	0.06	0.30	0
19	0	0	0	0	0	0	0.04	0	0	0.08	0	0.32	0.13	0.34	0.18	0.11	0.07	0.24	0	0	0.21	0	0	0.39	0
20	0	0	0	0	0	0.04	0	0	0	0	0	-0.18	0.38	0.66	0	0	0.20	0.47	0	0	0.22	0.25	0	0.30	0
21	-0.08	0	0	0.11	0	0	0	0	0	0	0.10	0.10	0	0	0.36	0.38	0.24	0	0.21	0.22	0	0.53	0.27	0.61	0
22	0	0.02	0	0	0	0	0	0	0	0	0	0.34	0.08	0.07	0.14	0.29	0.02	0.24	0	0.25	0.53	0	0.31	0.49	0
23	0	0	0	0	0	0.03	0	0	0	0.09	0	0.11	0	0.17	0	0.11	0	0.06	0	0	0.27	0.31	0	0.54	0
24	0	0	0.34	0	0	0	0	0	0	0.03	0	0.13	0.10	0.17	0	0	0.26	0.30	0.39	0.30	0.61	0.49	0.54	0	0
25	0.39	0.24	0	0	0	0	0.12	0	0	-0.07	-0.17	-0.07	0	0	0	0	0	0	0	0	0	0	0	0	0

*Node labels*: 1 = age of cannabis use initiation, 2 = lifetime cumulative frequency of cannabis use, 3 = childhood abuse, 4 = childhood neglect, 5 = urban upbringing, 6 = panic, 7 = anxious, 8 = sad, 9 = loss interest, 10 = irritable, 11 = manic, 12 = spying/following you, 13 = poison/hurt you, 14 = reading your mind, 15 = hear your thoughts, 16 = hear others thought, 17 = controlled by force, 18 = others stole thoughts, 19 = special messages/tv, 20 = hypnotized/magic/force, 21 = saw visions, 22 = heard noise/voice, 23 = smells/body odors, 24 = feelings in/on body, 25 = age at assessment.

*Childhood abuse* has positive connections with *feelings in/on body* (*w* = 0.34) and *spying/following you* (*w* = 0.09) from the psychosis dimension, and also has positive associations with the majority of affective symptoms, including *panic* (*w* = *0.18*), *sad* (*w* = 0.16), *anxious* (*w* = 0.11), and *irritable* (*w* = 0.07). *Childhood neglect* connects several psychotic experiences, i.e., *poison/hurt you* (*w* = 0.30)*, saw visions* (*w* = 0.11)*,* and *spying/following you* (*w* = 0.06), as well as to *loss interest* (*w* = 0.10) from the affective dimension. Effects of urbanicity on psychopathology were fully mediated by age of cannabis use initiation.

Stability analyses suggest that the network and identified edges are overall stable. Of the 121 identified edges, 114 (94.2%) were included in at least 50% of the bootstrapped network models. Of the edges connected to *age of cannabis use initiation* and *lifetime cumulative frequency*, all edges were included in at least 50% of the bootstrapped network models, except for the edge connecting *age of cannabis use initiation* with *childhood abuse*, indicating that this association should be interpreted with caution (see Supplementary Fig. 1). For the network showing stable edges only, see Supplementary Fig. 2. 59.5% of the participants could be left out to retain a correlation of *r* = 0.70 with the edge weights in the original model (see Supplementary Fig. 3), suggesting high stability of the results to dropping of cases^42^. All network connections related to the two variables representing cannabis use characteristics were retained at higher degrees of regularization (see Supplementary Fig. 4).

In the sex-moderated mixed graphical model, there was evidence that the association between *age of cannabis use initiation* and *age at assessment* was stronger in women than in men. Moreover, the association between *lifetime cumulative frequency of cannabis use* and *urbanicity* was stronger in men. However, results from bootstrapping suggest that these moderation effects were unstable, i.e., susceptible to sampling variation, and should be interpreted with caution.

## Discussion

We employed a data-driven network approach to explore the complex dependencies between cannabis use characteristics, i.e., age of initiation and lifetime cumulative frequency, and a broad spectrum of psychotic experiences and affective psychopathology as well as further early risk factors, i.e., childhood trauma and urban upbringing, in a general population sample. This approach allowed us to disentangle specific effects of age of initiation and lifetime cumulative frequency of cannabis use, while controlling for all other variables under consideration. There were three key findings: First, lifetime cumulative frequency of cannabis use showed particularly pronounced positive associations with delusional experiences, i.e., thought broadcasting and persecutory delusions, and, to a smaller extent, with auditory hallucinatory experiences. Second, age of cannabis use initiation showed negative associations with visual hallucinatory experiences and irritability, suggesting that these experiences become more likely the earlier use is initiated. Third, early risk factors, i.e., urban upbringing and childhood neglect, were stably linked to an earlier initiation, but not lifetime frequency of cannabis use. Results were stable and edges were overall estimated with good accuracy, and consistent across different levels of regularization.

The present study adds to a large body of evidence showing that early and frequent cannabis use do not only increase risk for full-blown psychotic symptoms observed in psychotic disorders, but also for psychotic experiences in non-clinical populations^9,20,22,24,39,71^, in line with a psychosis-proneness-persistence-impairment model of psychotic disorder^72^. Importantly, our results suggest that early and frequent cannabis use may have different relationships with different types of psychotic experiences. Replicating previous findings^24^, we find particularly pronounced associations of frequency of cannabis use with delusional experiences, especially persecutory ideas. Extending previous findings, we show that these particularly pronounced associations cannot be explained by age of cannabis use initiation. Collectively, these results underscore a differential association of frequency of cannabis use with hallucinations and delusions in the longer term that mirrors findings from acute cannabis intoxication^24,73,74^. In contrast, we found earlier cannabis use to be specifically associated with visual hallucinatory experiences. There was also a strong link between the two cannabis use characteristics in the network: Earlier cannabis use was associated with more frequent lifetime cannabis use. In line with previous epidemiological research, this pattern of results suggest that earlier initiation of cannabis use appears to be a key risk factor for vulnerability to the harmful psychopathological effects of cannabis use^8,9,11,15,20,22,75^ and increased, potentially problematic cannabis use later in life^12,76–78^. Thus, our findings corroborate the notion that delaying initiation of cannabis use is an important harm reduction intervention in terms of preventing or reducing later cannabis use and psychopathology^8,9,20,76,78^. This body of evidence is highly relevant from a public health perspective as the age of cannabis use initiation decreases and jurisdictions move toward legalization of cannabis^79,80^. Cannabis use in adolescence has been suggested to alter the development of various neurobiological systems^11,13–18^. Speculatively, early cannabis use may increase the risk for visual hallucinatory experiences by inducing lasting alteration in brain structures and functioning that serve the integration process of bottom-up perceptual information and prior expectations^27,81^. In accordance with this idea, previous research has shown that in patients with psychosis, cannabis use was linked to altered functional connectivity in visual attentional brain networks, and strength of connectivity was positively associated with a history of visual hallucinations, as well as a compound measure of cannabis use behaviors featuring earlier initiation^82^. While our findings do not allow conclusions about the underlying neurobiological mechanisms involved in the links between early cannabis use and visual hallucinatory experiences, they can serve as an informative intermediate step in a larger chain of interdisciplinary research efforts.

The present analysis also suggested links between earlier initiation of cannabis use and irritability. This finding highlights the relevance of specific affective experiences in cannabis-related psychopathology that were previously either not modeled^21,22^ or only assessed by sum scores^9,20^. Affective psychopathology has been suggested to play a key role in mediating between external triggers, such as cannabis use, and delusional ideas^28,40,83^. Consistent with this idea, increases in negative affect and perceptual aberrations fully explained increases in persecutory ideas following experimental administration of Δ9-tetrahydrocannabinol (THC), the main psychoactive component of cannabis , in a previous study^73^. Future work needs to carve out mechanistic pathways that account for the association between earlier initiation of cannabis use and individual affective symptoms, as well as their role in psychotic experiences.

Moreover, only earlier initiation, but not lifetime cumulative frequency of cannabis use was linked to early risk factors included in the network, i.e., experiences of childhood neglect and urbanicity. Interestingly, earlier initiation of cannabis use mediated the influence of urbanicity, a complex proxy environmental influence, on psychopathology, extending previous research that showed that lifetime cannabis exposure mediated effects between urbanicity and psychopathology in the past two weeks^25^. Our findings add specificity to this previously identified association, suggesting that earlier initiation, but not increased lifetime cumulative frequency of cannabis use, seems to become more likely given urban upbringing. Overall, these results might imply that psychogenic effects of growing up in urban surroundings may be partially explained by an earlier age of cannabis use initiation, which could, among other factors, be attributable to greater local availability of cannabis in non-rural areas compared to rural areas in the US^84,85^. This putative mechanism has important implications for public health, pointing to urban adolescent populations as a target for preventive campaigns of early cannabis use. Mirroring previous findings^12^, we also found early cannabis use to be associated with increased psychosocial risk in the form of childhood trauma, particularly neglect. It could be speculated that reduction of parental neglect might have a positive impact in terms of delaying initiation of cannabis use with the potential to prevent or reduce future cannabis use and psychotic experiences. Interestingly, cannabis use characteristics and childhood trauma showed unique as well as shared network links to specific psychotic experiences; specifically, visual hallucinatory experiences and persecutory delusions were associated with cannabis use as well as childhood trauma variables. This pattern of results may reflect both independent and additive pathways from environmental risk factors to specific psychotic experiences^36,37^. Overall, our findings underscore the complex interplay between different environmental risk factors, and that, when possible, they should be modeled jointly to assess their unique and shared effects on individual aspects of psychopathology^25,29,37,38^.

There are several limitations of the present study that need to be considered. First, given the cross-sectional nature of our data, reverse mechanisms, whereby psychotic or affective experiences in adolescence drive earlier initiation of cannabis use, reflecting self-medication or inclination towards risk-behaviors, cannot be excluded. Even though converging evidence based on longitudinal and retrospective designs renders this possibility rather unlikely^2,20,75,86^, analyses of prospective data are required to determine how earlier initiation of cannabis use maps onto individual affective and psychotic experiences through late adolescence and early adulthood. Similarly, inclusion of polygenic risk scores into the network may shed light on potential gene × environment interactions—for example, to assess to what extent genetic vulnerability may influence links between earlier initiation of cannabis use and psychopathology^24,26,87–91^. Second, older participants, on average, started using cannabis later than younger participants. This may reflect known historical cohort trends in age of cannabis use initiation^46,92^; however, biases in reports of early cannabis use due to recall error, social acceptance, and fear of disclosure may also play a role^46^. Third, the data used for modeling were collected in the 1990s. Since then, use patterns of cannabis have changed, especially with regard to harmful high-potency variants of cannabis products that have become increasingly available and popular in recent years^2,6,93,94^. In particular, there may be a role for frequent use of high potency variants of cannabis that we could not examine with the present data. Against the backdrop of ongoing debates^32^ and methodological advances in the network community^95^, the assessment of the replicability and generalizability of the present findings to diverse samples and present circumstances will be an important step for future research. Similarly, further samples may help to elucidate the role of additional factors, such as initiation age of tobacco smoking. Lastly, frequency of cannabis use was assessed in a binned format, which inevitably entails a loss of information. In general, quantification of drug use is a challenging task, with exact measures of the number of lifetime use occasions, especially in case of frequent use, likely being unreliable due to memory biases. Here, implementation of recently proposed minimum standards for quantifying cannabis use could facilitate the collection and integration of evidence on cannabis use across studies and disciplines^96^.

In conclusion, we employed a network approach to comprehensively explore unique associations between cannabis use characteristics, i.e., lifetime frequency and age of cannabis use initiation, and psychotic and affective psychopathology in a large, general population sample of cannabis users (i.e., those who reported having used cannabis at least once in their lifetime), while controlling for early risk factors and age at assessment. We found particularly pronounced associations between increased frequency of cannabis use and specific delusional experiences, i.e., persecutory delusions and thought broadcasting on the one hand, and earlier initiation of cannabis use and visual hallucinatory experiences and irritability on the other hand. Early risk factors were linked to an earlier initiation, but not frequency of cannabis use. Overall, these findings suggest that cannabis use characteristics may contribute differentially to risk for specific psychotic experiences and affective symptoms in the general population. Thus, we provide a valuable starting point for further investigation of the complex relationships between cannabis use patterns and specific symptoms.

## Supplementary Information

Supplementary Information.
