# Supplementary material for: A network approach to relationships between cannabis use characteristics and psychopathology in the general population
Source: Sci Rep. 2022 May 3;12:7163. doi: 10.1038/s41598-022-11092-0 (PMC9065088; doi:10.1038/s41598-022-11092-0)
Supplement: Supplementary file 1 — Supplementary Information. [file 41598_2022_11092_MOESM1_ESM.docx]

**Supplementary Material:**

**A network approach to relationships between cannabis use characteristics and psychopathology in the general population**

Linda T. Betz, Nora Penzel, Joseph Kambeitz

**Supplementary Figure 1.** Values of edges related to cannabis use characteristics (age of cannabis use initiation, lifetime cumulative frequency of cannabis use) with 95% confidence intervals obtained from bootstrapping.

**Supplementary Figure 2.** Network of cannabis use characteristics (age of cannabis use initiation, lifetime cumulative frequency of cannabis use), early risk factors, psychotic experiences, and affective symptoms (N = 2,544), including only edges which were present in at least 50% of the models generated based on non-parametric bootstrapped samples.

**Supplementary Figure 3.** Stability of edge weights obtained by case-dropping subset bootstrap.

**Supplementary Figure 4**. Network of cannabis use characteristics (age of cannabis use initiation, lifetime cumulative frequency of cannabis use), early risk factors, psychotic experiences, and affective symptoms (N = 2,544) across a range of reasonable values for gamma (0, 0.05, … 0.25).

**Supplementary Table 1.** Summary statistics for network variables along with relevant identifiers and corresponding node numbers plotted in the network.


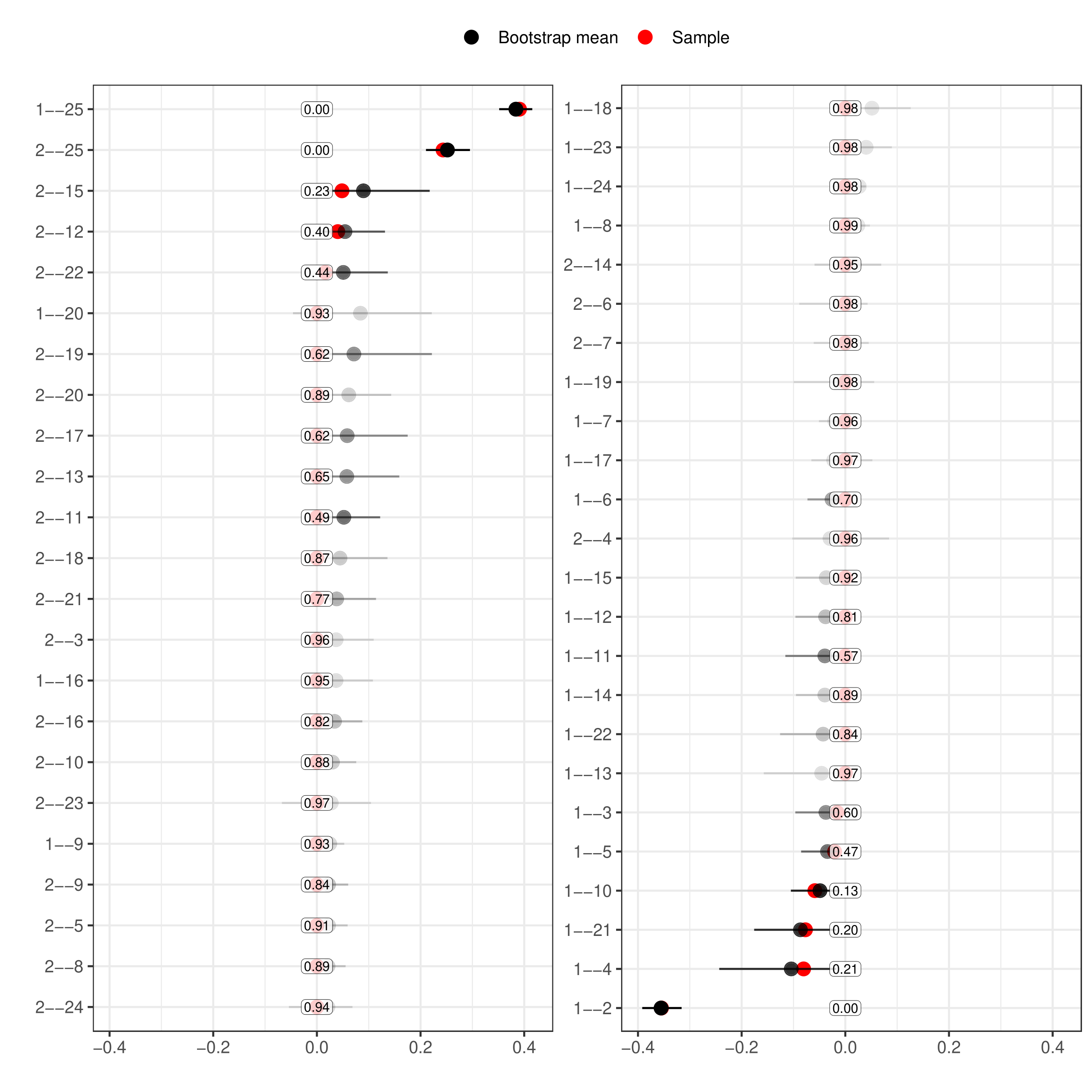


**Supplementary Figure 1.** Values of edges related to cannabis use characteristics (age of cannabis use initiation, lifetime cumulative frequency of cannabis use) with 95% confidence intervals obtained from bootstrapping. Confidence intervals are calculated based on those networks in which the edge was included (rather than set to zero). The transparency of the confidence interval reflects how often the edge was included in the networks generated in the bootstrapping procedure. The number in the box gives the proportion of sampled networks in which each edge was not included (i.e., set to zero). *Node labels*: 1 = age of cannabis use initiation, 2 = lifetime cumulative frequency of cannabis use, 3 = childhood abuse, 4 = childhood neglect, 5 = urban upbringing, 6 = panic, 7 = anxious, 8 = sad, 9 = loss interest, 10 = irritable, 11 = manic, 12 = spying/following you, 13 = poison/hurt you, 14 = reading your mind, 15 = hear your thoughts, 16 = hear others thought, 17 = controlled by force, 18 = others stole thoughts, 19 = special messages/tv, 20 = hypnotized/magic/force, 21 = saw visions, 22 = heard noise/voice, 23 = smells/body odors, 24 = feelings in/on body, 25 = age at assessment.

**
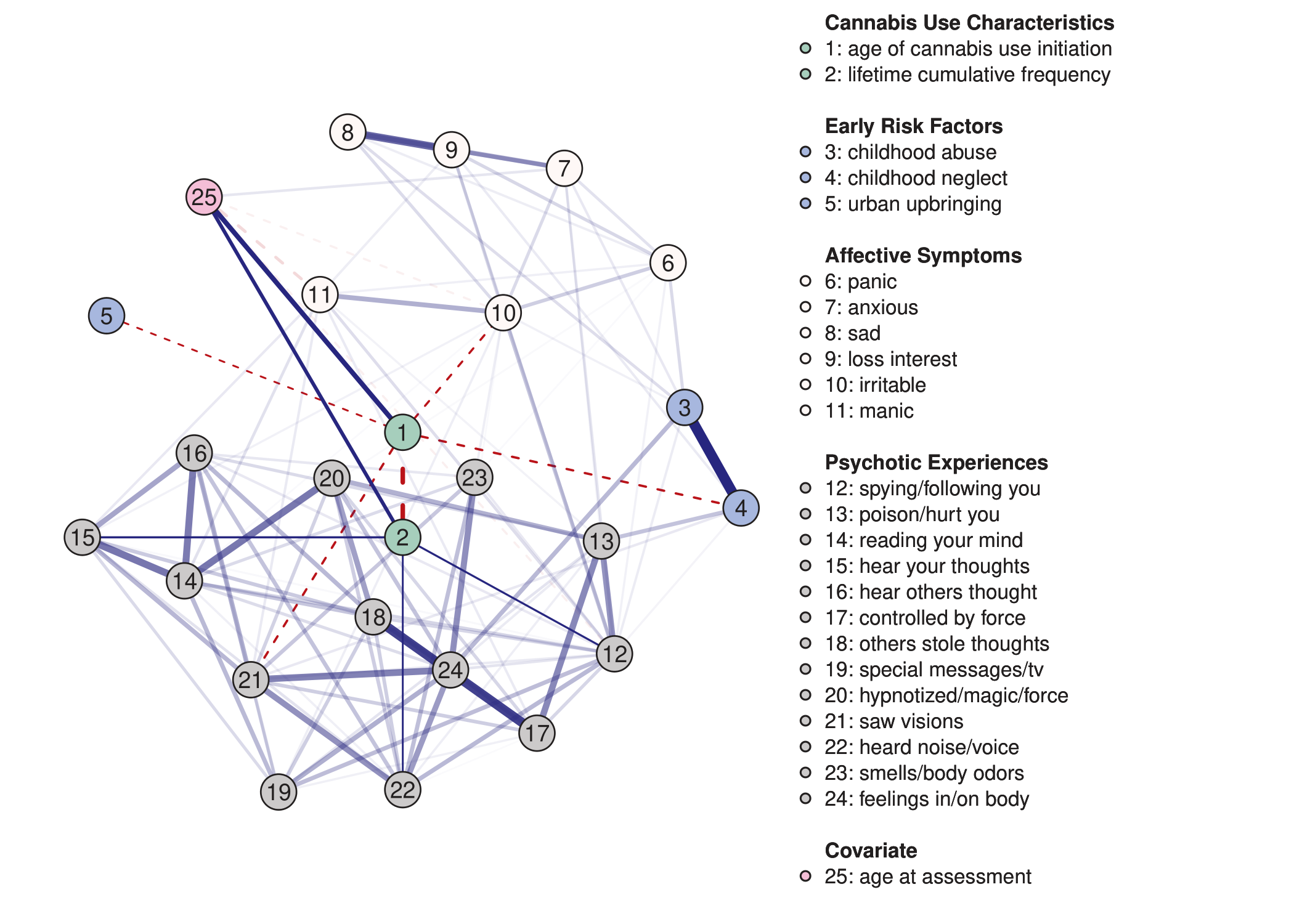
**

**Supplementary Figure 2.** Network of cannabis use characteristics (age of cannabis use initiation, lifetime cumulative frequency of cannabis use), early risk factors, psychotic experiences, and affective symptoms (N = 2,544), including only edges which were present in at least 50% of the models generated based on non-parametric bootstrapped samples. Solid blue (dashed red) lines represent positive (negative) associations between variables and wider, more saturated edges indicate stronger associations. Given that the focus of the paper is to investigate the relations between the cannabis use characteristics and aspects of psychopathology, the edges connecting to the two relevant variables (age of cannabis use initiation, lifetime cumulative frequency of cannabis use) have been manually un-faded, i.e., we set these edges deliberately opaque, while the edges between the other nodes in the network retain transparency. Variable groups are differentiated by color.


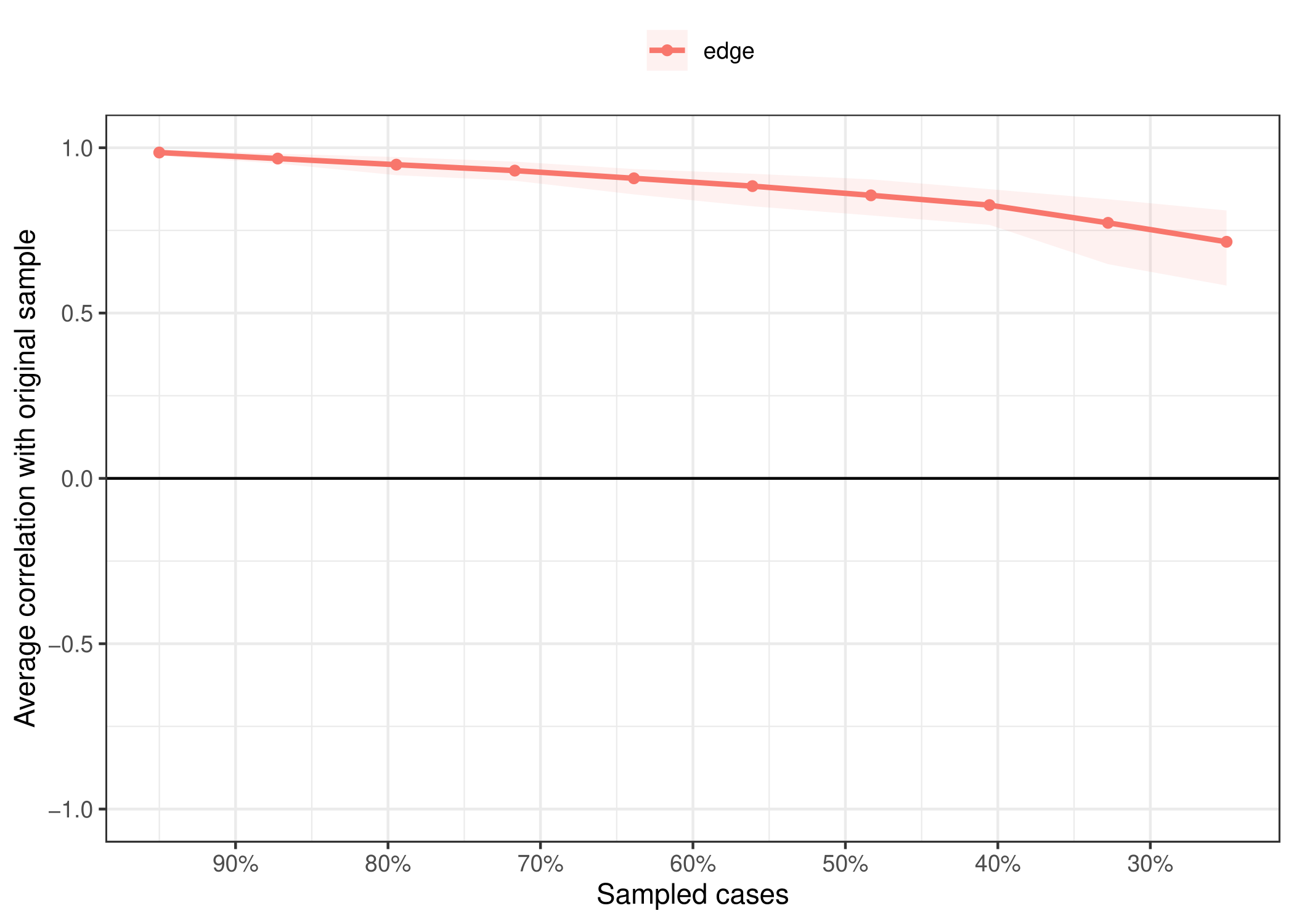


**Supplementary Figure 3.** Stability of edge weights obtained by case-dropping subset bootstrap^1^. The x-axis depicts the percentage of cases of the sample used at each step. The y-axis depicts the average of correlations between the edge weights from the original network and the edge weights from networks that were re-estimated after dropping increasing percentages of cases. The maximum proportion of observations that could be dropped while confidently (95%) retaining results that correlate highly (*r* > .7) with the edge weight estimates in the original sample was 59.5%, indicating high stability^2^.


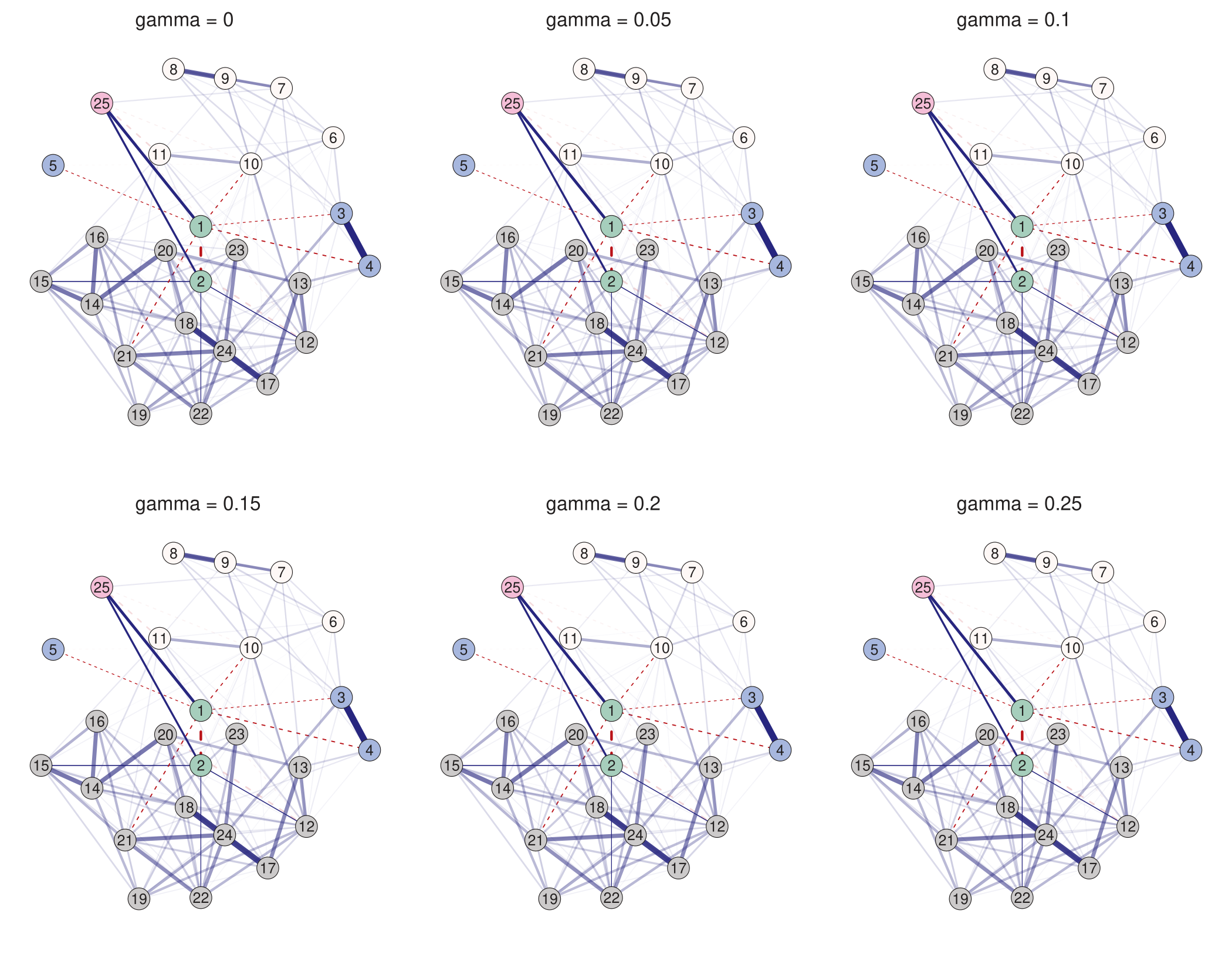


**Supplementary Figure 4**. Network of cannabis use characteristics (age of cannabis use initiation, lifetime cumulative frequency of cannabis use), early risk factors, psychotic experiences, and affective symptoms (N = 2,544) across a range of reasonable values for gamma (0, 0.05, … 0.25). The higher gamma, the higher the amount of regularization imposed on the network^2^. *Node labels*: 1 = age of cannabis use initiation, 2 = lifetime cumulative frequency of cannabis use, 3 = childhood abuse, 4 = childhood neglect, 5 = urban upbringing, 6 = panic, 7 = anxious, 8 = sad, 9 = loss interest, 10 = irritable, 11 = manic, 12 = spying/following you, 13 = poison/hurt you, 14 = reading your mind, 15 = hear your thoughts, 16 = hear others thought, 17 = controlled by force, 18 = others stole thoughts, 19 = special messages/tv, 20 = hypnotized/magic/force, 21 = saw visions, 22 = heard noise/voice, 23 = smells/body odors, 24 = feelings in/on body, 25 = age at assessment.

**Supplementary Table 1.** Summary statistics for network variables along with relevant identifiers and corresponding node numbers plotted in the network.

| **Network Variable** | **Question** | **Node** |  |
| --- | --- | --- | --- |
| *Cannabis Use Characteristics* |  |  |  |
| Age of cannabis use initiation (mean, SD) | How old were you the first time you used marijuana or hashish? | 1 | 16.7 (3.2) |
| Lifetime cumulative frequency (median) | About how many times in your life have  you used marijuana or hashish? | 2 | 11 to 49 times |
| *Early Risk Factors* |  |  | **% yes** |
| Childhood abuse | *See details in the method section.* | 3 | 16.6 |
| Childhood neglect | You were seriously neglected as a child (yes/no). | 4 | 4.6 |
| Urban upbringing | Was the area where you were raised during most of your childhood rural, a small town, a medium-sized town, a suburb, or a city? *(details in method)* | 5 | 46.4 |
| *Affective Symptoms* |  |  | **% yes** |
| Panic | Have you ever in your life had a spell or attack when all of a sudden you felt frightened, anxious or very uneasy in situations when most people would not be afraid or anxious? | 6 | 35.4 |
| Anxious | Have you ever had a period of one month or more when most of the time you felt worried or anxious? | 7 | 52.6 |
| Sad | In your lifetime, have you ever had two weeks or more when nearly every day you felt sad, blue, or depressed? | 8 | 54.3 |
| Loss interest | Has there ever been two weeks or more when you lost interest in most things like work, hobbies, or things you usually liked to do for fun? | 9 | 50.2 |
| Irritable | Has there ever been a period of several days when you were so irritable that you threw or broke things, started arguments, shouted at people, or hit someone? | 10 | 36.0 |
| Manic | Has there ever been a period of at least two days when you were so happy or excited that you got into trouble, or your family or friends worried about it, or a doctor said you were manic? | 11 | 11.7 |
| *Psychotic Experiences* |  |  | **% yes** |
| Spying/following you | Have you ever believed that people were spying on you or following you? | 12 | 14.3 |
| Poison/hurt you | Have you ever believed that you were being secretly tested or experimented on, that someone was plotting against you, or that someone was trying to poison you or hurt you? | 13 | 3.9 |
| Reading your mind | Have you ever believed that someone was reading your mind? | 14 | 7.8 |
| Hear your thoughts | Have you ever believed that others could hear your thoughts? | 15 | 4.5 |
| Hear others thought | Have you ever believed you could actually hear what another person was thinking, even though that person was not speaking? | 16 | 7.5 |
| Controlled by force | Have you ever been convinced that you were under the control of some power or force, so that your actions and thoughts were not your own? | 17 | 3.8 |
| Others stole thoughts | Have you ever been convinced that strange thoughts, or thoughts that were not your own, were being put directly into your mind, or that someone or something could steal your thoughts out of your mind? | 18 | 2.7 |
| Special messages/tv | Have you ever believed that you were being sent special messages through television or the radio, or that a program had been arranged just for you alone? | 19 | 2.7 |
| Hypnotized/magic/force | Have you ever felt strange forces working on you, as if you were being hypnotized or magic was being performed on you, or you were being hit by laser beams or X-rays? | 20 | 1.3 |
| Saw visions | Have you ever had the experience of seeing something or someone that others present could not see -- that is, had a vision when you were wide awake? | 21 | 9.0 |
| Heard noise/voice | Have you ever had the experience of hearing things that other people could not hear, such as noises or a voice? | 22 | 8.6 |
| Smells/body odors | Have you ever been bothered by strange smells around you that nobody else was able to smell, perhaps even odors coming from your own body? | 23 | 5.0 |
| Feelings in/on body | Have you ever had unusual feelings inside or on your body, like being touched when nothing was there or feeling something moving inside your body? | 24 | 8.5 |

**Supplementary References**

1. Costenbader, E. & Valente, T. W. The stability of centrality measures when networks are sampled. *Soc. Networks* **25**, 283–307 (2003).

2. Epskamp, S., Borsboom, D. & Fried, E. I. Estimating psychological networks and their accuracy: A tutorial paper. *Behav. Res. Methods* **50**, 195–212 (2018).
